# Outcomes of reverse total shoulder arthroplasty in patients ≤65 years old

**DOI:** 10.1016/j.jsea.2026.100041

**Published:** 2026-06-01

**Authors:** Kyle K. Obana, James Y. Chen, Doria L. Weiss, Andrew J. Luzzi, Michael L. Knudsen, Charles M. Jobin, William N. Levine

**Affiliations:** a Department of Orthopedic Surgery, Columbia University Irving Medical Center, New York, NY, USA; b Department of Orthopedic Surgery, NewYork-Presbyterian Hospital/Columbia University Medical Center, New York, NY, USA; c Investigation Performed at Columbia University Irving Medical Center, New York, NY, USA

**Keywords:** Young, Age, Shoulder surgery, Arthroplasty, Complications, rTSA, Outcomes

## Abstract

**Background:**

Reverse total shoulder arthroplasty (rTSA) is growing in popularity due to advancements in implant design, improvements in surgical techniques, and expanded indications. Hesitancy to perform rTSA in younger patients is due to concerns regarding implant longevity and functional outcomes. This study investigates outcomes of rTSA in patients ≤65 years old in the largest known cohort to date.

**Methods:**

Patients who underwent rTSA by 3 surgeons (C.M.J., M.L.K., W.N.L.) from 2014 to 2025 were retrospectively identified. Exclusion criteria were >65 years old, prior rTSA, and <12 months follow-up. Patient demographics, complications, and clinical outcomes were collected.

**Results:**

One hundred three patients were included. The average age was 59.5 ± 6.5 years and the average follow-up duration was 31.4 ± 27.2 months. Fifty six percent of patients had prior ipsilateral shoulder surgery. The most common diagnosis was cuff tear arthropathy (62.1%). Pre-operatively, 93.2% patients were independent. Post-operatively, 58.3% of patients were discharged the same day and 98.1% of patients went home. Pre-operative forward flexion was 81 ± 48°, external rotation at the side (ER side) was 16 ± 22°, ER at 90° abduction ER 90°) was 53 ± 24°, and internal rotation (IR) was L5. At 2-years post-operatively, forward flexion improved to 146 ± 20 (*P* < .01), ER side to 34 ± 14° (*P* < .01), ER 90° abduction to 81 ± 8° (*P* < .01), and IR to L3 (*P* < .01). Eight (7.8%) patients developed a post-operative complication that required revision surgery. Such complications occurred at an average of 25.8 ± 15.4 months post-operatively, with baseplate failure (37.5%) being the most common.

**Conclusion:**

rTSA can reliably improve clinical outcomes in patients ≤65 years old. Overall, complication rates are low despite most patients having had prior ipsilateral shoulder surgery.

Reverse total shoulder arthroplasty (rTSA) is a nonanatomic reconstruction of the glenohumeral joint that reliably improves shoulder function and pain. The procedure was initially reserved for older- and lower-demand patients due to initial rTSA iterations demonstrating early implant failure, limited range of motion (ROM) and complication rates up to 50%.^15^^,^^17^^,^^21^^,^^53^ However, complication rates have declined as surgical techniques have become more refined and implant designs have improved.^13^^,^^29^^,^^43^^,^^50^^,^^52^

Recently, clinical indications warranting rTSA in younger patients have expanded, resulting in greater utilization.^27^ Limited long-term durability of traditional implants posed a challenge when considering rTSA in younger patients with longer life expectancies and greater functional requirements. Given the historical role of rTSA as a salvage procedure with limited revision options, a large proportion of long-term data on rTSA in younger patients is biased toward cases with prior failed shoulder surgery and older prostheses, limiting meaningful subgroup analyses.^6^^,^^50^ Given these results, as well as imposed restrictions to avoid implant wear, younger patients may express concerns regarding post-operative function, limitations, and mobility relative to a native shoulder following rTSA.^31^^,^^41^ However, newer implant designs and refined surgical techniques have increased the longevity of rTSA implants, leading to more frequent use over anatomic TSA.^28^^,^^33^^,^^48^^,^^54^

Recent studies have reported promising results for rTSA in younger patients, with younger patients attaining greater ROM and similar patient-reported outcomes, pain score improvements, and complication rates compared to older patients.^6^^,^^11^^,^^22^^,^^29^^,^^33^^,^^34^^,^^36^^,^^50^ With younger patients reliably achieving improved activity and functional status following rTSA, a 333% increase in rTSA in younger patients is expected from 2011 to 2030.^11^^,^^16^^,^^44^^,^^47^^,^^50^ Although the few studies published on rTSA in younger patients demonstrate promising outcomes, these studies are limited in sample size.^51^ This study investigates outcomes of rTSA in patients ≤65 years old in the largest single center cohort to date.

## Materials and Methods

This study received institutional review board approval from the Columbia University Institutional Review Board. All patients who underwent rTSA (primary, conversion, revision) by 3 surgeons (C.M.J., M.L.K., W.N.L.) from 2014 to 2025 were retrospectively identified. Patient demographics were recorded, including age, baseline independence, sex, and comorbidities. Perioperative data were collected, including prior surgeries, diagnosis/indication for revision, implant type (Arthrex, DJO, Smith & Nephew, Stryker, and Zimmer Biomet), intraoperative blood loss, intraoperative complications, and final disposition.

ROM was recorded pre-operatively and at six months, 1, two, and three years post-operatively. Clinical ROM included forward flexion (FF), external rotation at the side (ER side), ER at 90° shoulder abduction (ER 90°), and internal rotation (IR). Post-operative complications were defined as any implant- or surgical-related complications that were managed nonoperatively or required reoperation. Radiographic review assessed any complications (eg, change in implant position/loosening, fracture, osteolysis, scapular notching, stress shielding). Exclusion criteria were >65 years old, prior rTSA, and <12 months follow-up. Follow-up was defined as a clinical visit documenting ROM following surgery.

### Statistical analysis

Continuous variables were reported as means, standard deviations, and ranges. Chi-squared and Fisher exact tests were used to evaluate categorical variables and risk factor assessments. Paired student t-tests were used to compare pre-operative to post-operative ROM. Statistical analyses were performed using STATA/MP Software (version 13.0; StataCorp LLC, College Station, TX). Statistical significance was set at *P* < .05.

## Results

One hundred eighty seven patients who underwent rTSA and were ≤65 years old were retrospectively identified. Out of these, 103 (55.1%) had ≥1 year follow-up and were included in the study. The average age was 59.5 ± 6.5 years (range 27 to 65 years) and the average follow-up duration was 31.4 ± 27.2 months (Table I). Fifty six percent of patients had prior ipsilateral shoulder surgery with a mean of 1.8 ± 1.1 surgeries (range 1 to 5 surgeries). Prior surgeries included rotator cuff/labral repair (69.0%), arthroplasty (15.5%), open reduction internal fixation (10.3%), and Latarjet/distal tibial allograft (5.2%) (Table II). The 3 most common diagnoses were cuff tear arthropathy (CTA) (62.1%), glenohumeral arthritis without rotator cuff tear (9.7%), and proximal humerus fracture (8.7%) (Table III). Implant systems used for surgery were 63 (61.2%) Zimmer Biomet, 27 (26.2%) DJO, 7 (6.8%) Smith & Nephew, 5 (4.9%) Stryker, and 1 (1.0%) Arthrex.

**Table I:** Patient demographics.

Patient demographics	%
Males	52.4%
Age (yr)	59.6 ± 6.4 yr [27, 65]
Follow-up (months)	31.4 ± 27.2 mo [12.1, 137.7]
≥2 yr	46.6%
≥3 yr	30.1%
≥4 yr	20.4%
Number of prior surgeries	1.8 ± 1.1 surgeries [1, 5]
Prior shoulder surgery	56.3%
Dominant side operated	63.1%
Hypertension	50.5%
Diabetes mellitus	12.6%
Heart disease	10.7%
Osteoporosis	3.9%
Smoking status	
Never	62.1%
Former	30.1%
Current	7.8%
Functional status	
Independent	93.2%
Family/home health aid	5.8%
Nursing home	1.0%

**Table II:** Prior shoulder surgeries.

Prior shoulder surgery	%
Rotator cuff/labral repair	69.0
Arthroplasty (resurfacing, HA, aTSA)	15.5
ORIF	10.3
Latarjet/DTA	5.2

*aTSA*, anatomic total shoulder arthroplasty; *DTA*, distal tibial allograft; *HA*, hemiarthroplasty; *ORIF*, open reduction internal fixation.

**Table III:** Diagnosis warranting reverse total shoulder arthroplasty.

Diagnosis	%
CTA	62.1
Glenohumeral arthritis	9.7
PHFx	*8.7*
Failed TSA	*6.8*
Failed ORIF	*4.9*
Instability	3.9
Inflammatory arthritis	1.9
AVN	1.0
Failed HA	1.0

*AVN*, avascular necrosis; *CTA*, cuff tear arthropathy; *HA*, hemiarthroplasty; *ORIF*, open reduction internal fixation; *PHFx*, proximal humerus fracture; *TSA*, total shoulder arthroplasty.

Pre-operatively, 93.2% of patients were completely independent, 5.8% required support of family or a home health aide, and 1.0% resided at a nursing facility. Following surgery, 58.3% of patients went home the same day and the remaining 41.7% were admitted for an average of 1.4 ± 0.6 nights (Table IV). Final disposition included 98.1% of patients going home and 1.9% to a subacute rehabilitation facility.

**Table IV:** Hospital course following reverse total shoulder arthroplasty including disposition, complications, and inpatient stay.

Hospital course	%
Outpatient	58.3%
Inpatient stay	1.4 ± 0.6 days [1, 3]
Intraoperative complications	0%
Inpatient complications	0%
Final disposition	
Home	98.1%
Subacute rehabilitation	1.9%

Pre-operative (n = 103) FF was 85 ± 46° (95% confidence interval [CI]: 75 to 94°), ER side was 16 ± 22° (95% CI: 10 to 18°), ER 90° was 53 ± 24° (95% CI: 47 to 59°), and IR was L5 (95% CI: L4 to S1) (Table V). At six months post-operatively (n = 69), FF improved to 146 ± 21° (95% CI: 141 to 151°; *P* < .01), ER side to 36 ± 12° (95% CI: 32 to 39°; *P* < .01), ER 90° abduction to 83 ± 9° (95% CI: 79 to 87°; *P* < .01), and IR to L3 (95% CI: L2 to L4; *P* < .01). At two years (n = 30) post-operatively, FF improved to 146 ± 20° (95% CI: 139 to 153°; *P* < .01), ER side to 34 ± 14° (95% CI: 28 to 40°; *P* < .01), ER 90° abduction to 81 ± 8°(95% CI: 77 to 85°; *P* < .01), and IR to L3 (95% CI: L2 to L5; *P* < .01).

**Table V:** Pre-operative vs. post-operative range of motion following reverse total shoulder arthroplasty in patients ≤65 years old.

ROM	Pre-operative (˚) n = 103	6 mo post-op (˚) n = 69	1 y post-op (˚) n = 77	2 y post-op (˚) n = 30	3 y post-op (˚) n = 18
FF	85 ± 46 [0, 170]	**145** ± **23 [90, 180]**	**149** ± **21 [90, 180]**	**146** ± **20 [90, 170]**	**145** ± **22 [90, 180]**
ER side	14 ± 19 [−30, 70]	**36** ± **12 [5, 60]**	**40** ± **14 [0, 80]**	**34** ± **14 [10, 60]**	**43** ± **14 [20, 70]**
ER 90°	53 ± 24 [0, 90]	**83** ± **9 [60, 90]**	**83** ± **13 [30, 90]**	**81** ± **8 [60, 90]**	**84** ± **8 [70, 90]**
IR	L5, [T7, side]	**L3, [T7, side]**	**L3, [T5, side]**	**L3, [T9, side]**	**L2, [T9, side]**

*ROM*, range of motion; *FF*, forward flexion; *ER*, external rotation; *IR*, internal rotation; post-op, post-operative.

Bolded values = significantly improved from pre-operatively (*P* < .05).

Results presented as mean ± standard deviation, range [#, #].

There were 11 (10.7%) total complications in this study. Eight (7.8%) patients developed a post-operative complication that required revision surgery. Such complications occurred at an average of 25.8 ± 15.4 months post-operatively, with baseplate failure (37.5%) being the most common. (Table VI). Additional complications treated nonoperatively were 1 patient with an acromial stress fracture (1.0%), 1 patient with a greater tuberosity nonunion (1.0%), and 1 patient with scapular notching to the inferior baseplate screw (1.0%). No statistically significant associations were detected between complications and prior ipsilateral shoulder surgery, prior open shoulder surgery, sex, smoking status, diabetes, osteoporosis, or implant type.

**Table VI:** Complications requiring revision surgery.

Complications	n (%)
Baseplate failure	3 (37.5)
Instability	2 (25.0)
Polyethylene wear	2 (25.0)
Scapular notching	1 (12.5)
Total	8 (100)

## Discussion

This study demonstrates significant improvement in post-operative ROM and few complications following rTSA in patients ≤65 years of age. Importantly, patients demonstrated significant improvements in FF, ER, and IR at six months post-operatively, which surpass clinical thresholds to perform activities of daily living and are associated with patient satisfaction.^38^^,^^39^ ROM was maintained through three years post-operatively, consistent with prior studies.^19^ Although this study did not analyze return to sport or activities following rTSA, patients can be reassured that there is a high rate (85.5%) of returning to at least 1 sport following rTSA, with 88.2% reporting sports outcome as good to excellent.^18^ However, PROMs and functional outcomes in young patients following rTSA can be heterogenous, as some studies report similar outcomes compared to that of elderly patients while others report poorer outcomes, attributed to greater pre-operative function in younger patients.^32^ Neel et al found that young patients who underwent rTSA demonstrated lower satisfaction and functional outcomes compared to that of older patients despite similar post-operative ROM.^40^ This may be attributed to greater expectations of post-operative function and higher baseline functional status.

Complication rates and implant longevity are important considerations in younger patients with longer life expectancies.^10^ Baseplate failure, instability, and polyethylene wear were the most common causes for revision surgery in this study, consistent with prior reports.^7^^,^^20^^,^^26^^,^^50^ Instability is a more common cause of revision surgery following rTSA in younger patients relative to older patients.^24^^,^^26^ Although it is unclear why younger patients demonstrate higher rates of instability, it is hypothesized that reasons may include higher activity levels, greater functional demands, and higher rates of previous shoulder surgeries.^2^^,^^40^ Importantly, the overall complication rate requiring reoperation in the current study was 7.8%, which is at the lower end of rates reported in prior studies (7% to 39%).^3^^,^^6^^,^^7^^,^^13^^,^^14^^,^^29^^,^^50^ Neither prior ipsilateral shoulder surgery nor prior open ipsilateral surgery was associated with greater risk of revision surgery in the current study, which contrasts with prior reports.^8^^,^^52^ The authors acknowledge that this study does not predict or support the long-term survivorship of rTSA implants in young patients. However, since instability risk is greatest during the first 2 post-operative years, it is possible that complication rates during the 31.4 month average follow-up duration in this study may be informative of longer-term follow-up.^5^^,^^9^^,^^20^^,^^45^^,^^46^

To the author's knowledge this is the first study to report disposition of young patients following rTSA. Most patients in this study were completely independent prior to surgery (93%) and almost all were discharged home (98%). This contrasts with older patients undergoing rTSA, who demonstrate a higher propensity for hospital admission (77%), longer inpatient stay (3 to four days), and greater need for subacute rehabilitation or acute rehabilitation (25% to 54%).^1^^,^^42^ Furthermore, the impact of the COVID-19 pandemic has led to an increase in discharge-home and decreased hospital length of stay.^25^ Despite the high pre-operative function and independence, 42% of patients in this study required overnight stay in the hospital despite 93% being independent pre-operatively. This is important, as various factors such as pain, anesthesia, timing of surgery, and home support may preclude same day discharge even in healthy, independent individuals.^35^ Patients should be counseled that although they may require an overnight stay, they can be expected to return to their original living situation.

Similarly, there is no consensus regarding what defines a “young” patient who undergoes rTSA. The youngest patient included in the current study was 27 years old at the time of surgery, while the oldest was 65 years old. Given the natural history of rotator cuff degeneration with progressing age, patients closer to 65 years old are more likely to have diagnoses of CTA relative to younger patients, who often have alternative diagnoses such as chronic instability with severe glenoid bone loss or prior shoulder arthroplasty. An age cutoff of ≤65 years old was selected in accordance with prior literature, although some studies have used a threshold of ≤60 years old.^6^^,^^14^^,^^38^^,^^40^^,^^50^ Chronological age alone is an imperfect guide for surgical decision making. Comorbidities, prior surgeries, motivation, activity demand, and overall physiological health are factors that may influence post-operative outcomes more so than chronological age following rTSA.^12^^,^^23^^,^^30^^,^^37^

Expectedly, over half of the patients in the current study had prior ipsilateral shoulder surgery, which is consistent with studies on younger patients undergoing rTSA (56% to 83%).^6^^,^^13^^,^^38^^,^^40^^,^^50^ Additionally, the average number of prior surgeries was 1.8 surgeries (range: 1 to 5), which is similar to that of prior reports (1.6 to 2.5 surgeries).^6^^,^^50^ This can be attributed to the overall complexity of shoulder pathologies in the younger demographic (eg, soft tissue and bony instability surgeries, multiple failed acute rotator cuff repairs, significant glenoid bone loss, proximal humerus fracture) necessitating rTSA as a salvage procedure. Comparatively, a smaller proportion of older patients receiving primary rTSA have had prior ipsilateral shoulder surgery (15.4% to 26%).^4^^,^^42^^,^^49^

There are multiple limitations in this study. The first limitation is the broad age of inclusion in this study (≤65 years old), with the youngest patient being 27 years old at the time of surgery. As mentioned previously, indications for rTSA in patients ≤65 years of age are broad, and surgeons should consider pre-operative functional status, comorbidities, prior surgeries, and physiological health to guide patient expectations. Second, this study is a retrospective case series and is therefore limited by the availability and completeness of data within the medical record. Collection of PROMs was not performed routinely in clinic, so these outcomes were not presented in this study. Similarly, the authors did not have multiple individuals evaluating ROM in clinic to assess interrater reliability. To address these limitations, the authors are conducting a prospective study to collect long-term outcomes. Third, only 77 (74.8%) of patients had 1 year follow-up and 30 (29.1%) had two year follow-up. Loss to follow-up is likely attributable to multiple factors and may result in under-representation or over-representation of post-operative complications in the current study. However, reported clinical outcomes and post-operative complications are similar to those of prior studies with longer follow-up.^6^^,^^7^^,^^11^^,^^29^^,^^52^ Ongoing prospective data collection is intended to increase sample size, allow systematic collection of PROMs, and better elucidate the long-term prognosis of young patients undergoing rTSA. Fourth, this cohort is heterogeneous based on prior surgeries and diagnoses. Subgroup analyses were not performed as the sample size within each limited further analysis. Larger cohorts and multicenter studies may be able to detect differences that exist between patients with CTA, fracture, instability, and revision/conversion cases. Lastly, it is important to note that the 3 surgeons in this study are all shoulder/elbow-fellowship trained, and each performs ≥200 shoulder arthroplasties per year. Thus, these outcomes may not reflect that of the general population.

## Conclusion

rTSA can reliably improve ROM in patients ≤65 years old. Overall, complication rates are low despite most patients having had prior ipsilateral shoulder surgery. Almost all patients were discharged home to their pre-operative living situations.

## Disclaimers:

Funding: No funding was disclosed by the authors.

Conflicts of interest: The author, their immediate family, and any research foundation with which they are affiliated have not received any financial payments or other benefits from any commercial entity related to the subject of this article.
